# The protective role of jervine against radiation-induced gastrointestinal toxicity

**DOI:** 10.1080/14756366.2019.1586681

**Published:** 2019-03-14

**Authors:** Selvinaz Yakan, Tuba Aydin, Canan Gulmez, Ozkan Ozden, Kivilcim Eren Erdogan, Yusuf Kenan Daglioglu, Fundagul Andic, Onur Atakisi, Ahmet Cakir

**Affiliations:** a Animal Health Department, Agri Ibrahim Cecen University Eleskirt Celal Oruc School of Animal Production, Agri, Turkey;; b Faculty of Pharmacy, Agri Ibrahim Cecen University, Agri, Turkey;; c Department of Pharmacy Services, Tuzluca Vocational School, Igdir University, Igdir, Turkey;; d Department of Bioengineering, Faculty of Engineering and Architecture, Kafkas University, Kars, Turkey;; e Department of Pathology, Faculty of Medicine, Cukurova University, Adana, Turkey;; f Experimental Medicine Research Center, Cukurova University, Adana, Turkey;; g Department of Radiation Oncology, Faculty of Medicine, Cukurova University, Adana, Turkey;; h Department of Chemistry, Faculty of Science and Letter, Kafkas University, Kars, Turkey;; i Department of Chemistry, Faculty of Science and Literature, Kilis 7 Aralık University, Kilis, Turkey

**Keywords:** Radiotherapy, jervine, pyruvate dehydrogenase (PDH)

## Abstract

In this study, we investigated whether jervine (J) could prevent gastrointestinal (GI) side effects of abdominopelvic radiotherapy (RT) in Wistar-Albino female rats. Rats were divided into five groups: control (C), J only (J), J administered at 5 mg/kg/days for 7 days, RT only (RT), J before RT (J + RT), J administered for seven days before RT, J both before and after RT (J + RT + J), and J administered for 7 days before RT and after RT for 3 days. The weights of rats were measured on the 1st, 7th, and 10th days of the study. Rats were sacrificed to obtain tissues from the liver and intestine, which was followed by taking blood samples intracardially. In addition, the tissues were stained with pyruvate dehydrogenase (PDH) immunohistochemically. In our study, J supplementation markedly reduced weight loss, and histopathological, immunohistochemical, biochemical results suggest that J had a protective effect on GI toxicity following RT.

## Introduction

Radiotherapy (RT) means treating cancer using ionising radiation^1–4^. The high dose of radiation given by RT can kill cancer cells and prevent them from dividing and multiplying. However, RT causes injury to healthy tissues close to the target tissue by direct or indirect mechanisms. The injury spectrum ranges from acute self-limiting reaction to life-threatening complications^5–8^. Gastrointestinal (GI) toxicity in patients with irradiated GI tracts may cause severe nutritional problems and significant weight loss, impairing patient feeding. Injured GI tissue again epithelised causes dehydration and malnutrition. These concerns may cause RT to be interrupted, causing RT to terminate prematurely, and tumour control and survival rates may be reduced by preventing administration of higher doses of RT^9–11^. Therefore, the use of radioprotective agents in the control of acute radiation tissue injury is prominent. Various agents are used to reduce the side effects of normal tissue without altering the tumoural effect of RT. Experimental and clinical studies were performed with radioprotective agents such as amifostine, sucralfate, pentoxifylline, prostaglandin synthesis inhibitors, hematopoietic cytokines, and antioxidants^11^. However, although the efficacy of many agents has been investigated to prevent tissue injury due to radiation, there is still a need for an effective, side-ineffective, easy-to-use radioprotector for reducing side effects of RT^11–13^. Jervine (J) ((*2*′*R, 3S, 3*′*R, 3*′*aS, 6*′*S, 6aS, 6bS, 7*′*aR, 11aS, 11bR*)-2,3,3′a,4,4′,5′,6,6′,6a,6b,7,7′, 7′a,8,11a,11b-Hexadecahydro-3-hydroxy-3′,6′,10,11b-tetramethyl-spiro[9H-benzo[a] fluorene-9,2′(3′H)-furo [3,2-b] pyridin]-11(1H)-one)) is one of the steroidal alkaloids isolated from *Veratrum album*^14^. Steroidal alkaloid-rich extracts have been reported to be tested against some cancer cells and have anticancer properties^15–17^. J is reported to have antitumour activity^18^, and in another study on J, it was reported that it is a potent antioxidant and has an anti-inflammatory effect^19^.

We aimed to research whether J could prevent acute GI side effects of abdominopelvic RT in Wistar-Albino rats.

## Materials and methods

This research was approved by the Cukurova University Animal Experiments Local Ethics Committee (approval number: 2018-01/04). Thirty-five Wistar-Albino rats with the following conditions were included: raised at a temperature of 22 ± 2 °C, fed standard rat feed and tap water, aged 2.5–3 months, 200 ± 5 g in weight, female, and healthy. Each group had seven rats randomly divided into five groups: control (C), J only, RT only, J before RT (J + RT), and J before and after RT (J + RT + J). The first group was the C group. The second group was administered only J and RT was not administered. The third group was administered RT alone. The fourth group was administered J before RT. The fifth group was administered J both before and after RT.

### Preparation of plant extract

The undervalued parts of the *Veratrum album* were ground in the blender without drying directly in the sun. Then, 1300×*g* of ground material was treated with 7.5% (3.4 mol) of diluted NH_4_OH and then extracted with benzene (5 L) at 40 °C in a reflux cooler. The extract was filtered, and the filtrate was concentrated by rotary evaporator at 50 °C and low pressure. At the end of this process, 24 g of the extract was obtained^19^.

### Experimental procedure

To rats in the J groups (that J, J + RT, J + RT + J groups) were fed J by gastric gavage at 5 mg/kg/day per morning in addition to their daily nutrients in the same laboratory conditions. The control group was administered water with gastric gavage. J was administered for 7 days to the J group. J was administered for seven days before RT to the J + RT group, J was administered for seven days before RT and three days after RT to the J + RT + J group (three days following RT).

The rats were sedated with 10 mg/kg xylazine hydrochloride (Alfazyne^®^, %2, Alfasan International, 3440 AB, Woerden, Holland) and 50 mg/kg ketamine (Ketalar^®^, Pfizer Pharma GMBH, Berlin, Germany) intraperitoneal injection prior to RT. RT was performed before at least 6 h after J was administered. The rats' abdominopelvic regions were irradiated at 8 Gray (Gy) in a single fraction protecting the head and thoraxes from antero-posterior (A/P) using 6 MV X-ray energy with a Low Energy Varian Clinac 600C DBX (Varian Medical Systems, Palo Alto, CA). A 0.5 cm bolus was used during the treatment.

The weight of the rats was measured on the 1st, 7th, and 10th days of the experiment. In J, J + RT, and J + RT + J groups, weight measurements were performed before J was administered. After all of these applications, the rats were sacrificed to obtain the liver and intestine following the collection of intracardiac blood samples in the J group on the 7th day of the experiment and on the 10th day for other rat groups. Table 1 shows the timing of weight measurements by group.

**Table 1.** Procedure timing for all groups^a^.

	Groups									
C										
J	J	J	J	J	J	J	J			
RT							RT			
J + RT	J	J	J	J	J	J	J + RT			
J + RT + J	J	J	J	J	J	J	J + RT	J	J	J
Days	1^b^	2	3	4	5	6	7^b^	8	9	10^b^

^a^C: control; J: jervine; RT: radiotherapy; J + RT: J before RT; J + RT + J: J before and after RT group.

^b^Weight measurements.

### Histopathological evaluation

After sacrificing the rats, intestines and livers were dissected. Macroscopically, the intestines were sampled from at least three different areas, especially for damaged areas, to evaluate tissue healing. Liver tissues were sectioned serially and evaluated for abnormal findings. Tissues were fixed in 10% formaldehyde, and paraffine-embedded blocks were cut (4–5 µm) and stained with haematoxylin–eosin using an automatic staining device (Leica ST5020, Wetzlar, Germany). Tissues were evaluated for degenerative changes under light microscopy, such as crypt abscess, congestion, intraepithelial lymphoid infiltration, and inflammation.

The degenerative change was scored. For crypt abscess, grade 0 no crypt abscess was observed, grade 1 was a sparse crypt abscess formation, grade 2 was less than 50% crypt abscess formation, and grade 3 was more than 50% crypt abscess formation. For congestion assessment, grade 0 no congestion was observed, grade 1 was less than 20% congestion, grade 2 was more than 20% but less than 50% congestion, and grade 3 was over 50% congestion observed. The number of intraepithelial lymphocytes was counted among 100 surface epithelial cells to score the lymphoid infiltration and counted as the number of inflammatory cells among 100 surface epithelial cells for intraepithelial inflammation^5^.

### Immunohistochemical analysis

The intestinal tissues were fixed in buffered 10% formalin and then processed for standard (5 μm) paraffin sections. Then, sections were incubated for 30 min in 3% H_2_O_2_, and nonspecific binding was blocked with normal serum. The PDH was incubated with primary antibodies against PDH overnight at 4 °C. The same concentration of normal serum served as a negative control. The bound antibodies were eventually detected with the biotin-streptavidin peroxidase system. Then, samples were incubated with 3,3′-diaminobenzidine (DAB) peroxidase substrate solution, and the slides was counterstained with haematoxylin. The immunostained images were captured using a digital camera microscope (Nikon Digital Sight DS-L3, Tokyo, Japan).

### Determination of biochemical parameters

Serum total protein concentration was determined by the Bradford^20^ method using bovine serum albumin (BSA) as the standard at 595 nm^21–23^. Serum albumin and globulin level, alanine aminotransferase (ALT), aspartate aminotransferase (AST), and gamma-glutamyl transferase (GGT) activity were determined spectrophotometrically using commercial kits.

### Statistical analysis

Weight measurements and statistical analyses of biochemical data were performed using Statistical Package for the Social Sciences (SPSS) Windows 16.0 packaged software by SPSS Inc. (SPSS Inc., Chicago, IL). Between-group average values were determined by one-way analysis of variance (ANOVA), and differences between groups were assessed with Duncan’s *post hoc* test. The results were reported as the mean and standard deviation (mean ± SD). In the analysis of the histopathological data, the 19.0 version of the SPSS statistical package was used for summary statistics (mean, standard deviation, minimum, and maximum), and cross tables were used for summarising categorical variables. In cross tables, the existence of a relationship between categorical variables was examined by chi-square tests. The similarity of the distribution was investigated with the Kruskal–Wallis tests for more than two groups. When differences were found, the Mann–Whitney *U* test was used for pairwise comparisons. *p* Values less than .05 were considered significant. Materiality levels were determined by the Bonferroni correction. *p* < .008 was considered significant for the evaluation of histopathological findings.

## Results

### Weight changes

No differences were observed among the groups on the 1st day of the experiment (*p* > .05). It was observed that the animals in the C group had the most increased weight among all animals during the 10 days of the experiment, but the rats who received RT (that RT, J + RT, and J + RT + J groups) lost weight compared to the 7th day of the experiment. On the 10th day of the last weight measurement, groups receiving RT lost weight compared to the C group: RT (*p*< .001), J + RT (*p*< .001), and J + RT + J (*p*< .001). When groups receiving RT were compared over the 10 days of the experiment, RT with J + RT (*p*> .05) and RT with J + RT + J (*p*> .05), there was no statistically significant difference, but the increase in weight was larger in groups receiving J (J + RT, J + RT + J). When J + RT and J + RT + J groups were compared, there were no statistically significant differences in weight increase (*p*> .05). Weight measurements by group and day are given in Table 2.

**Table 2.** Mean and standard deviation (mean ± SD) of weight changes between days 1 and 10 for all groups^a^.

	Weight (g)			
Group	1 day	7 days	10 days	*p*
C (*n*:7)	201.29 ± 0.521^x^	210.43 ± 0.611^y^	214.86 ± 0.340^z^	*p* < .001
J (*n*:7)	201.0 ± 0.577^x^	211.57 ± 0.571^y^	–	*p* < .001
RT (*n*:7)	200.86 ± 0.459^x^	210.71 ± 0.680^y^	204.43 ± 0.84^z,^^b^	*p* < .001
J + RT (*n*:7)	201.0 ± 0.577^x^	209.86 ± 0.670^y^	207.0 ± 0.308^z,^^b^	*p* < .001
J + RT + J (*n*:7)	200.97 ± 0.222^x^	210.43 ± 0.649^y^	208.57 ± 0.368^z,^^b^	*p* < .001
*p*	Ns	Ns	*p* < .001	

Differences are statistically significant within groups marked with different letters (x, y, z) on the same line (*p* < .001).

^a^C: control; J: jervine; RT: radiotherapy; J + RT: J before RT; J + RT + J: J before and after RT group.

^b^In the among groups comparison, there was a statistically significant difference between the values of different character in the same column (*p* < .05). Ns: none significant.

### Histopathological evaluation

**Figure 1.**
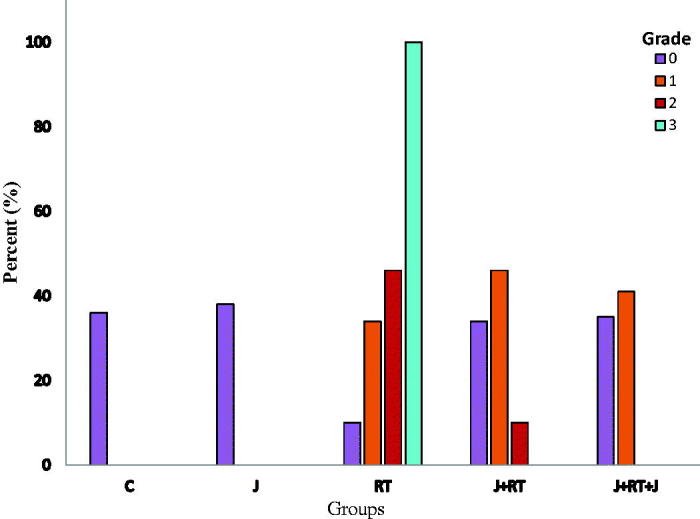
Subepithelial crypt abscess formation by group. C: control; J: jervine; RT: radiotherapy; J + RT: J before RT; J + RT + J: J before and after RT group.

All groups differed in terms of intraepithelial degenerative changes in crypt abscesses (grade 0, 1, 2, and 3) (*p*≤ .001) (Figure 1). To evaluate the severity of crypt abscesses, grades 0–1 and grades 2–3 crypt abscesses were compared among groups (Table 3). No grades 2 and 3 subepithelial crypt abscesses were observed in the C and J groups. There was no difference between C and J groups (*p*= 1). While 58.1% of grades 2 and 3 subepithelial crypt abscesses were seen in the RT group, this rate statistically significantly reduced 9.1% in the J + RT group (*p* = .001) and 0% in the J + RT + J group (*p*< .001). When J + RT and J + RT + J groups were compared, the presence of grades 2 and 3 subepithelial crypt abscesses reduced from 9.1% to 0%, and there was no statistically significant difference between groups (*p*= .428). When the RT group with and the groups not receiving RT (that C, J groups) were compared, the grades 2 and 3 subepithelial crypt abscesses in the RT group were statistically significant (*p*< .001). There was no statistically significant difference between the no RT group and the J + RT group (*p*= .321). Grades 2 and 3 subepithelial crypt abscesses were not seen in either group when comparing no the RT group and the J + RT + J group. When comparing the group not receiving RT and the J + RT + J group, the difference was no significant (*p*= 1) (Table 3) (Figure 5(A)).

**Table 3.** Distributions of grades 0–1 and grades 2–3 intraepithelial degenerative changes – subepithelial crypt abscess formation and congestion according to the groups^a^.

	Intraepithelial degenerative changes in subepithelial crypt abscess formation				Intraepithelial degenerative changes in congestion			
	Grades 0 and 1		Grades 2 and 3		Grades 0 and 1		Grades 2 and 3	
Group	*n*	%	*n*	%	*n*	%	*n*	%
C	7	100	0	0	7	100	0	0
J	7	100	0	0	7	100	0	0
RT	3	41.9	4	58.1	0	0	7	100
J + RT	6	90.9	1	9.1	6	90.9	1	9.1
J + RT + J	7	100	0	0	7	100	0	0

^a^C: control; J: jervine; RT: radiotherapy; J + RT: J before RT; J + RT + J: J before and after RT group.

**Figure 2.**
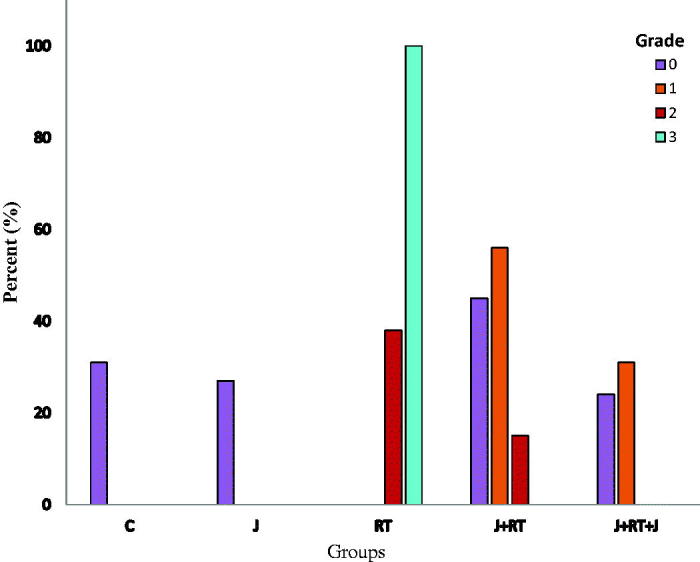
Intraepithelial degenerative changes in congestion by group. C: control; J: jervine; RT: radiotherapy; J + RT: J before RT; J + RT + J: J before and after RT group.

All groups differed in terms of intraepithelial degenerative changes in congestion (grades 0, 1, 2, and 3) (*p*≤ .001) (Figure 2). To evaluate the severity of intraepithelial degenerative changes in congestion, grades 0–1 and grades 2–3 degenerative changes in congestion were compared among groups (Table 3). There were no grades 2 and 3 degenerative changes in congestion in the C and J group. There was no difference between C and J groups (*p*= .441). While congestion was seen to be 100% grades 2 and 3 in the RT group, and this rate statistically significantly decreased to 9.1% in J + RT group (*p*< .001) and 0% in J + RT + J group (*p*< .001). When J + RT and J + RT + J groups were compared, the presence of grades 2 and 3 degenerative changes in congestion decreased from 9.1% to 0%, and there was no statistically significant difference between groups (*p*= .137). When the no RT group was compared with RT groups, the grades 2 and 3 degenerative changes in congestion in the RT group were statistically significant (*p*< .001). When the group not receiving RT was compared with the J + RT group, J + RT degenerative changes in congestion were higher, but the differences between groups were not statistically significant (*p*= .011). However, when the no RT group was compared with the J + RT + J group, the degenerative changes in congestion seen in the J + RT + J group was significantly reduced, and there was no difference between groups (*p*= .221) (Table 3) (Figure 5(B)).

**Figure 3.**
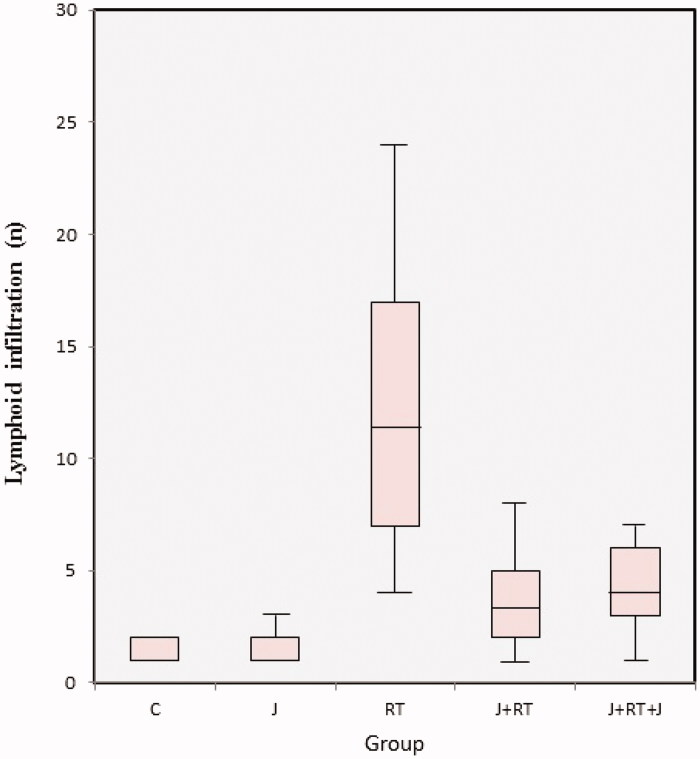
Intraepithelial lymphoid infiltration by group. C: control; J: jervine; RT: radiotherapy; J + RT: J before RT; J + RT + J: J before and after RT group.

All groups were different in terms of intraepithelial lymphoid infiltration (*p*≤ .001) (Figure 3). In Table 4, mean ± SD and median (minimum–maximum) values are given. When the C and J groups were compared, there was no difference between groups (*p*= .543). When the RT group was compared with the J + RT and J + RT + J groups, there was statistically significantly lower lymphoid infiltration in the J + RT (*p*< .001) and J + RT + J groups (*p*< .001). When J + RT and J + RT + J groups were compared, there was no statistically significant difference between groups (*p*= .448). When the no RT groups and RT groups were compared, lymphoid infiltration was statistically significantly higher in the RT group (*p*< .001). When the no RT group was compared with J + RT and J + RT + J groups, lymphoid infiltration was statistically significantly higher in J + RT and J + RT + J groups (*p*< .001) (Table 5) (Figure 5(C)).

**Table 4.** Distributions in histopathological results by group.

	None-RT	RT	J + RT	J + RT + J	*p*
Subepithelial grades 2–3 abscess formation*	0(0) and 0(0)	4(58.1)	1(9.1)	0(0)	<.001^a,^^d,^^e^
Intraepithelial grades 2–3 degenerative changes-congestion*	0(0) and 0(0)	7(100)	1(9.1)	0(0)	<.001^a,^^d,^^e^
Intraepithelial lymphoid infiltration**	0.57 ± 0.6, 0 (1–2) and 0.7 ± 0.8, 0 (1–3)	16.0 ± 2.8, 13 (4–24)	4.4 ± 1.0, 3 (0–8)	3.4 ± 0.8, 4 (0–7)	<.00^a,^^b,^^c,^^d,^^e^
Intraepithelial inflammation**	0.1 ± 0.2, 0 (0–1) and 0.1 ± 0.3, 0 (0–1)	2 ± 2.8, 4 (1–9)	1.3 ± 1.9, 3 (1–5)	0.6 ± 1.1, 2.5 (1–4)	<.001^a,^^d,^^e^

None-RT (C: control, J: jervine); RT (radiotherapy); J + RT (J before RT); J + RT + J (J before and after RT) group.

*n (%).

** Mean ± SD, median (minimum–maximum).

* Bonferroni’s adjustment applied for multiple comparisons, i.e. *p*= .05/6=.008 is accepted as significant.

^a^*p*< .008 for none-RT vs. RT.

^b^*p*< .008 for none-RT vs. J + RT.

^c^*p*< .008 for none-RT vs. J + RT + J.

^d^*p*< .008 for RT vs. J + RT.

^e^*p*< .008 for RT vs. J + RT + J.

**Table 5.** Comparison of histopathological results by group.

	None-RT vs. RT	None-RT vs. J + RT	None-RT vs. J + RT + J	RT vs. J + RT	RT vs. J + RT + J	J + RT vs. J + RT + J
Subepithelial grades 2–3 abscess formation	*p* < .001*	*p*=.321	*p* = 1	*p*=.001*	*p* < .001*	*p*=.428
Intraepithelial grades 2–3 degenerative changes-congestion	*p* < .001*	*p*=.011	*p*=.221	*p* < .001*	*p* < .001*	*p*=.137
Intraepithelial lymphoid infiltration	*p* < .001*	*p* < .001*	*p* < .001*	*p* < .001*	*p* < .001*	*p*=.448
Intraepithelial inflammation	*p* < .001*	*p*=.029	*p*=.179	*p* < .001	*p* < .001	*p*=.479

None-RT (C: control; J: jervine); RT (radiotherapy); J + RT (J before RT); J + RT + J (J before and after RT) group.

* Bonferroni adjustment applied for multiple comparisons, i.e. *p*= .05/6=.008 is accepted as significant.

**Figure 4.**
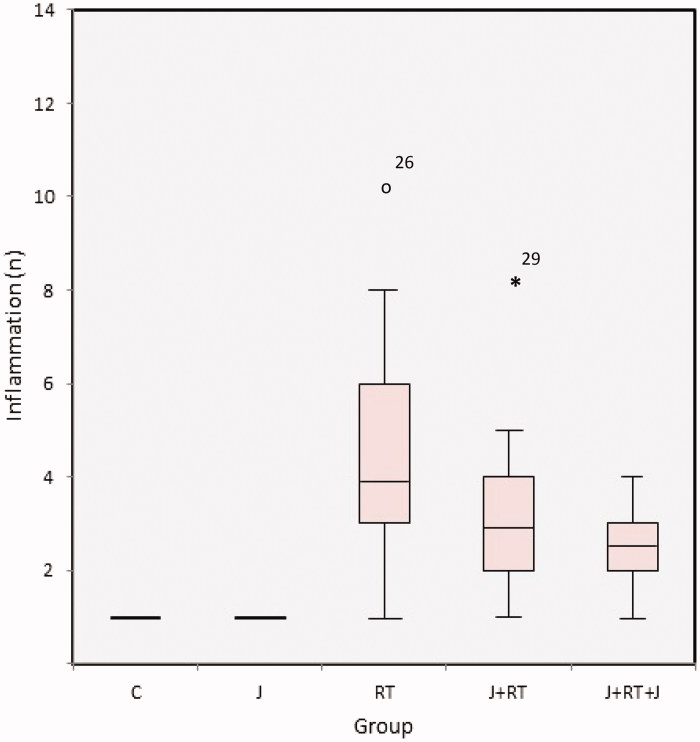
Intraepithelial inflammation by group. C: control; J: jervine; RT: radiotherapy; J + RT: J before RT; J + RT + J: J before and after RT group.

**Figure 5.**
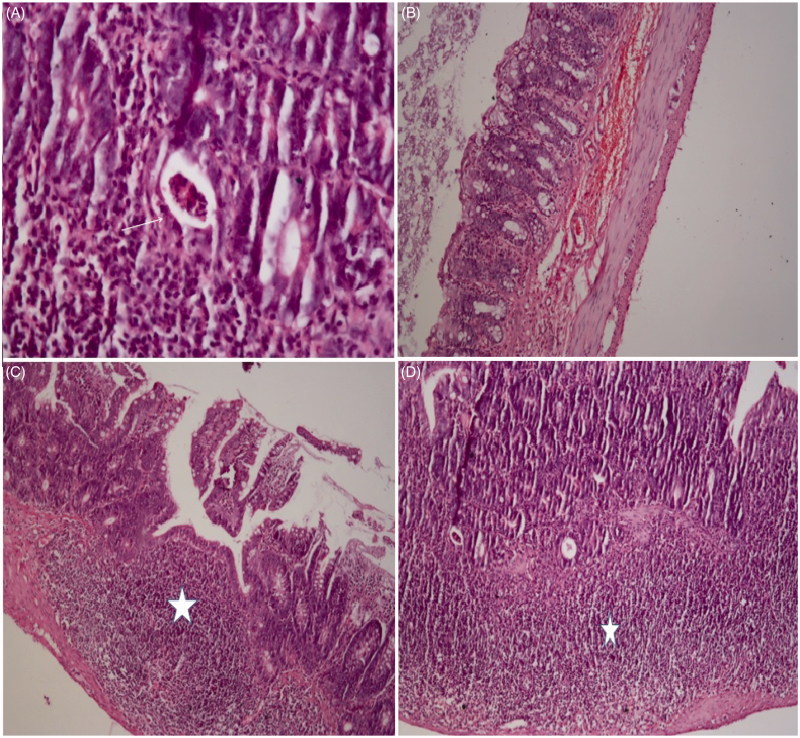
Intestine, radiation-induced histopathological changes. Histopathology is shown under the light microscope of the intestine using haematoxylin–eosin (H&E) in the rats that received RT. (A) Crypt abscess (arrow) in the small intestine, H&E ×200. (B) Congestion in the colon wall, H&E ×100. (C) Intensive lymphoid infiltration (star) in the small intestine H&E ×100. (D) Dense inflammation (star) in the small intestine, H&E ×100.

All groups were different in terms of intraepithelial inflammation (*p*≤ .001) (Figure 4). In Table 4, mean ± SD and median (minimum–maximum) values are given. When the C and J groups were compared, there was no difference between groups (*p*= .117). When the RT group was compared with J + RT and J + RT + J groups, there was statistically significantly lower intraepithelial inflammation in the J + RT (*p*< .001) and J + RT + J groups (*p*< .001). When J + RT and J + RT + J groups were compared, intraepithelial inflammation was less common in the J + RT + J group, but there was no statistically significant difference (*p*= .479). Comparing the no RT group with RT groups, intraepithelial inflammation in the RT group was statistically significant (*p*< .001). Comparing the no RT group with the J + RT group, the difference was not statistically significant between groups (*p*= .029). When the no RT group and the J + RT + J group were compared, the difference in inflammation was not statistically significant but the inflammation in the J + RT + J group was significantly decreased (*p*= .179) (Table 5) (Figure 5(D)).

### Immunohistochemical analysis results

All groups have views of intestinal tissues stained with PDH immunohistochemically in Figure 6(1,2). Immunostaining is not observed in Figure 6(1,A), which is small intestine tissue from the C group and (B), the colon tissue from the C group. Likewise, (C), small intestine tissue from the J group and (D), the colon tissue from the J group is shown. There was no observed change in PDH activity in intestinal tissues in the C and J groups (Figure 6(1)).

Figure 6.(1) Immunostaining of PDH was not observed in the C and J groups. (A), small intestine tissue in (×100) and (B), the colon tissue in C group (×100) likewise, (C), small intestine tissue (×100), and (D), the colon tissue in J group (×100). (2) The intensity of PDH staining significantly decreased in the intestine in J + RT and J + RT + J groups compared to the RT group. (A), RT group shows positive cytoplasmic PDH cells in small intestinal tissues (×100). (B), J + RT + J group does not show positive immunostaining in small intestinal tissues (×100). (C), RT group shows positive cytoplasmic PDH cells in colon tissues (×100). (D), J + RT group shows weak positive cytoplasmic PDH cells in colon tissues (×100).

**Figure:**
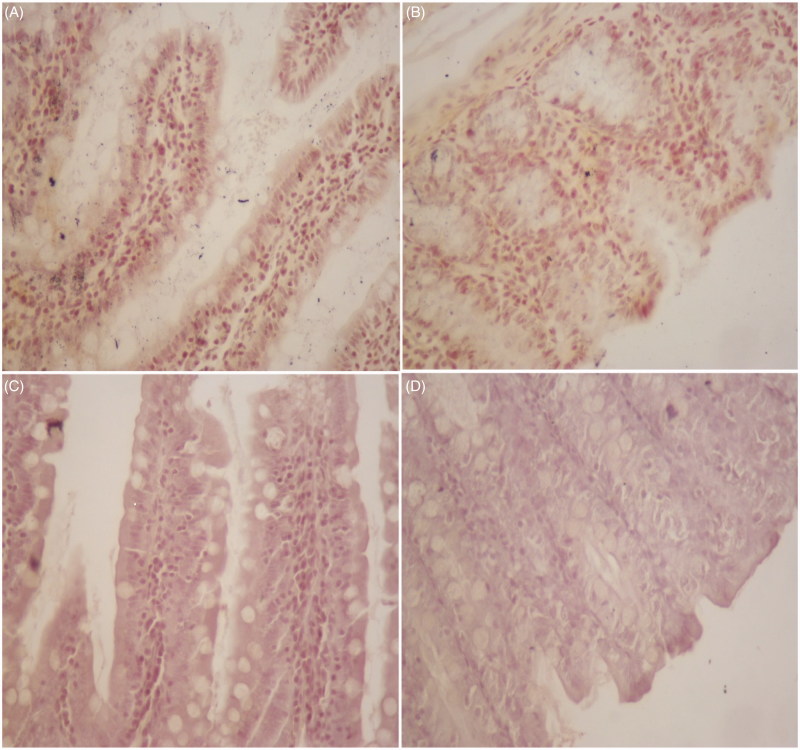

**Figure:**
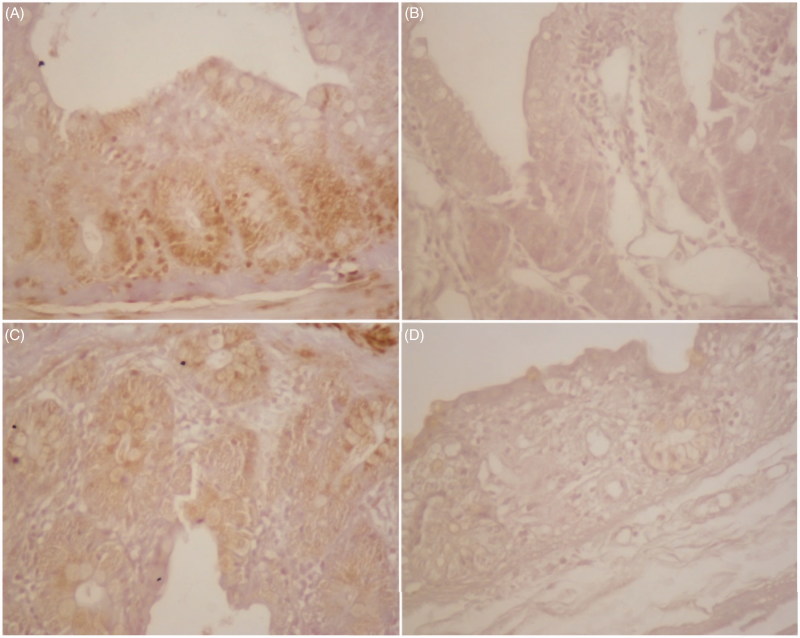

The RT group showed positive cytoplasmic PDH cells in small intestinal tissues compared to the C and J groups (Figure 6.2.A). Immunostaining was not observed in small intestinal tissues in the J + RT + J group compared to the RT group (Figure 6(2,B)). The RT group shows positive cytoplasmic PDH cells in colon tissues compared to the C and J groups (Figure 6(2,C)). The J + RT group shows weak positive cytoplasmic PDH cells in colon tissues compared to the RT group (Figure 6(2,D)).

### Biochemical results

There was no statistically significant difference in biochemical parameters in total protein, albumin, globulin, albumin/globulin levels among groups (*p*> .05). There was no statistically significant difference between the C and J groups in ALT activity (*p*< .05). When comparing RT and J + RT groups, there were no statistically significant differences between groups, although ALT activity was decreased in the J + RT group (*p*> .05). When comparing RT and J + RT + J groups, ALT activity decreased significantly significant in the J + RT + J group (*p*< .001). When J + RT and J + RT + J groups were compared, there was a statistically significant decrease in ALT activity in the J + RT + J group (*p*< .001). When the no RT and RT groups were compared, there was a statistically significant increase in the ALT activity in the RT group (*p*< .001). When the no RT and J + RT groups were compared, there was a statistically significant increase in ALT activity in the J + RT group (*p*< .001). When the no RT and J + RT + J groups were compared, the difference was not statistically significant between groups (*p*> .05). AST activity was not statistically significantly different compared between the C and J groups (*p*> .05). When the no RT and RT groups were compared, there was a statistically significant increase in AST activity in the RT group (*p*< .05). When the RT group was compared with the J + RT and J + RT + J groups, there was statistically significant decreased AST activity in the J + RT and J + RT + J groups (*p*< .05). Comparing the J + RT and J + RT + J groups, there was no statistically significant difference between groups (*p*> .05). There was no statistically significant difference in GGT levels among all groups (*p*> .05). The results of biochemical measurements are given in Table 6.

**Table 6.** Serum means values and standard deviation (*X*±SD) of biochemical parameters by group^a^.

	Groups					
	C	J	RT	J + RT	J + RT + J	*p*
Total protein (g/dL)	7.20 ± 0.045	6.87 ± 0.092	6.85 ± 0.076	7.10 ± 0.041	7.05 ± 0.090	Ns
Albumin (g/dL)	3.22 ± 0.068	3.04 ± 0.059	2.76 ± 0.239	2.78 ± 0.200	3.11 ± 0.081	Ns
Globulin (g/dL)	3.99 ± 0.056	3.83 ± 0.139	4.18 ± 0.244	4.32 ± 0.231	3.88 ± 0.188	Ns
Albumin/globulin	0.81 ± 0.029	0.80 ± 0.045	0.72 ± 0.104	0.66 ± 0.082	0.82 ± 0.071	Ns
ALT (U/L)	6.98 ± 0.023^y^	7.84 ± 1.295^y^	22.51 ± 3.602^x^	19.37 ± 4.86^x^	6.88 ± 0.761^y^	<.001
AST (U/L)	8.19 ± 1.766^y^	10.32 ± 3.80^y^	16.49 ± 1.305^x^	13.09 ± 1.511^xy^	13.67 ± 0.291^xy^	<.05
GGT (U/L)	1.31 ± 0.654	1.33 ± 0.078	2.63 ± 0.454	1.18 ± 0.382	1.30 ± 0.371	Ns

ALT: alanine aminotransferase; AST: aspartate aminotransferase; GGT: gamma glutamyl transfer; Ns: none significant.

Differences are statistically significant among groups marked with different letters (x, y) on the same line (*p* < .05, *p* < .001, respectively).

^a^C: control; J: jervine; RT: radiotherapy; J + RT: J before RT; J + RT + J: J before and after RT group.

## Discussion

Radioprotector agents have become an important research topic to protect normal tissues from the negative effects of radiation^5^. In previous studies, many agents have been used that are antioxidants (such as amifostine and glutamine), anti-inflammatory agents (such as entolimod), antiproliferative agents (such as curcumin), anti-apoptotic agents (such as silymarin), protectors of endothelial cells (such as vitamin D), prostaglandin synthesis inhibitors (such as benzydamine), and hematopoietic cytokines (such as IL-1 and TNF- α)^4^^,^^11^. Although the efficacy of many agents has been investigated for the prevention of tissue damage due to radiation, there is still a need for an effective, low side-effect, easy-to-use radioprotector for reducing the side effects of RT. An ideal radioprotective agent is considered one that is stable, easy to administer, preferably using oral administration, is available at low financial burden, does not reduce the antitumoural activity of RT and chemotherapy while maintaining normal tissues, does not cause treatment morbidity, has no life-threatening effects, and has no persistent toxicity. To date, no radioprotective agent with successful long-term clinical outcomes has been reported. Recently, for antioxidative effects, the interest in agents that reduce the damage caused by RT in normal tissue has increased^4^^,^^11^. Antioxidants react with free radicals and have antioxidative effects on DNA damage and cell membrane damage, prevent tissue damage and reduce radioprotective effects as well as having antimutagenic and anticarcinogenic properties^11^. Jervine, a steroidal alkaloid, was first isolated from the *Veratrum genus* in 1991^14^. J is one of the major steroidal alkaloids found among Veratrum species. J was reported to have antitumour activity, and it is also an analogue of cyclopamine, a major steroidal alkaloid^19^. Cyclopamine has been reported to inhibit the Hedgehog signalling pathway, which is important in the proliferation of cancerous cells. Due to this feature, extracts of steroidal alkaloids obtained from J and other *Veratrum* species have been tested against some cancer cells and have been reported to have anticancer properties^24^^,^^25^. In a study to determine the anti-inflammatory and antioxidant activity of J, all tested doses significantly prevented acute inflammation caused by carrageenan (CAR). CAR has been shown to significantly reduce cytokines in serum, neutrophil infiltration and lipid peroxidation in tissues. It has been shown that CAR has a negative effect on many antioxidant enzyme activities and GSH levels, and that J rebuilds the antioxidant defence system, reduces lipid peroxidation in tissues, reduces the level of cytokines in serum and reduces neutrophil infiltration. J potency was found to be anti-inflammatory and antioxidant^19^.

In this experimental study, we aimed to investigate whether or not J is effective at reducing radiation damage through anti-inflammatory and antioxidant mechanisms and to evaluate the acute side effects of RT and the protective effects of J on radiation damage in the intestine through clinical, histopathological, immunohistochemical, and biochemical measurements. J did not kill rats at the selected dose of 8 Gy RT, which was considered a moderate dose^11^. GI complications developed in patients receiving RT to abdominal and pelvic regions. The most common acute clinical side effects from abdominopelvic irradiation are lack of appetite, fatigue, abdominal pain, nausea, vomiting, and diarrhoea. Diarrhoea is the most common acute GI system toxicity^4^^,^^5^^,^^11^^,^^26^. In a study investigating the effects on nutritional status and side effects associated with treatment and quality of life for patients treated with hydroxy methyl butyrate/arginine/glutamine, weight changes, treatment side effects, malaise, malnutrition risk, skeletal muscle mass, prealbumin levels, and albumin levels improved, although the difference was not statistically significant. There was no significant difference between the control and treatment groups in terms of quality of life^27^. In a study that investigated the efficacy of lycopene in preventing radiation esophagitis, it was reported that lycopene had a radioprotective effect but lycopene addition to RT had no protective effect on weight loss^5^. In our study in rats, diarrhoea and weight loss symptoms were evaluated because symptoms such as fatigue, loss of appetite, abdominal pain, nausea, and vomiting could not be evaluated. No diarrhoea was observed in any of the cases in the group. Abdominopelvic RT-related weight loss may be due to the severity of intestinal damage caused by RT and may be due to nausea and loss of appetite as shown in Andic et al.^11^. In our study, by the 7th day of the experiment, the animals in all groups gained weight based on their normal development. On the 10th day of the experiment, it was observed that the groups receiving RT (RT, J + RT, and J + RT + J) were weaker than on the 7th day of the experiment. However, in our study, because of statistically significant greater weight gain in the J + RT and J + RT + J groups when compared to the RT group (*p*< .05), J addition to RT had a protective effect on weight loss. In this study, similar to Andic et al.^11^, weight loss in rats due to RT was attributed to side effects such as nausea, which could not be evaluated clinically (Table 2).

GI syndrome begins to develop three days after RT, which is the first time that signs of impaired mucosal integrity can be first seen histologically^11^^,^^28^. Free radicals from RT damage DNA and the cell cycle breaks down^11^. The damage and death of stem cells within the intestinal crypt in the G2 and M stages of mitosis lead to a decrease in the production of intestinal epithelial cells, and as a result, loss of mucosal integrity. Histopathologically, there is epithelial peeling and crypt micro-abscess formation as a result of the loss of stem cells within the intestinal crypt and mitosis of the crypt. Inflammatory cell infiltration is observed in the intestinal surface epithelium, lamina propria, and mucosa. Submucosal congestion may occur due to obstructive artery occlusion. Epithelial dysfunction may exacerbate mucosal inflammation, leading to an increase in the passage of intestinal pathogens^28–32^. In our study, we sacrificed rats three days after RT to evaluate histopathological changes in the intestine. Epithelial cells covering the intestine are mitotically very active. The pathogenesis of early period lesions is due to the direct effect of radiation on subepithelial cells^28^.

In our study, we evaluated the formation of crypt abscesses, congestion, lymphoid infiltration and inflammation from histopathological findings. In our study, the effect of J on crypt abscess formation was statistically significantly lower in the J + RT (58.1% and 9.1%, *p*= .001) and J + RT + J (58.1% and 0%, *p*< .001) groups compared to the RT group. When the J + RT group was compared with no RT groups, the grades 2 and 3 crypt abscesses in the J + RT group were slightly higher (0% and 9.1%, *p*= .321), but the difference was not statistically significant. However, no grades 2 and 3 crypt abscesses were observed in the no RT and J + RT + J groups (*p*= 1) (Figure 1) (Table 3). The intraepithelial grades 2 and 3 degenerative changes in congestion showed that in intestines in the J + RT group (100% and 9.1%, *p*< .001) and the J + RT + J group (100% and 0%, *p*< .001) were statistically significantly lower compared to the RT group. Comparing the J + RT group with no RT groups, although the grades 2 and 3 degenerative changes in congestion were higher in the J + RT group (0% and 9.1%, *p*= .0011), the difference was not statistically significant. When comparing the J + RT + J group with no RT groups, the intraepithelial degenerative changes in congestion in the J + RT + J group disappeared completely (0% and 0%, *p*= .221) and there were no differences between groups (Figure 2) (Table 3). Intraepithelial lymphoid infiltration in the J + RT group was statistically significantly (*p*< .001) greater than in no RT groups, but it was significantly lower than (*p*< .001) in the RT group. In the same way, intraepithelial lymphoid infiltration in the J + RT + J group was statistically significantly higher than the no RT groups (*p*< .001), but statistically significantly less than the RT groups (*p*< .001) (Figure 3) (Tables 4 and 5). Intraepithelial inflammation in the intestine was statistically significant and lower in both the J + RT group (*p*< .01) and the J + RT + J group (*p*< .01) compared to the RT group. Comparing J + RT with no RT groups, the J + RT group had more intraepithelial inflammation but the difference was not statistically significant (*p*= .029). Comparing the J + RT + J group with the no RT groups, intraepithelial inflammation in the J + RT + J group decreased significantly (*p*= .179). When J + RT and J + RT + J groups were compared, intraepithelial inflammation was less common in the J + RT + J group but there was no statistically significant difference (*p*= .479) (Figure 4) (Tables 4 and 5). When all histopathological parameters were evaluated, improvement in the J + RT + J group was better than the J + RT group. Therefore, it was thought that it would be more useful to give J before and after RT for radioprotection (Figure 5).

Impaired mitochondrial function is the result of overexpression of HIF-1-induced PDH which inactivates pyruvate dehydrogenase multi-enzyme complex (PDC) by adding a phosphate group to the TCA cycle by converting the pyruvate into acetyl-CoA^33^. HIF-1-induced PDH is caused by overexpression. HIF-1 stimulates enzymes and lactate dehydrogenase in glycolysis and induces pyruvate dehydrogenase kinase-1 (PDK1) in the mitochondria. PDK1 inhibits the activity of the pyruvate dehydrogenase multienzyme complex (PDH or PDC), which provides acetyl-CoA to the TCA cycle, and thus oxidative phosphorylation slows down. The slowing of oxidative phosphorylation also reduces the formation of reactive oxygen species (ROS). With this mechanism, HIF-1 in hypoxic conditions keeps ROS formation in balance. Otherwise, ROS-induced apoptosis may occur. The use of PDH inhibitors is a novel treatment strategy that directs oxidative phosphorylation from glycolysis of cancer cells, thus stimulating apoptosis^34–37^. In our study, cytoplasmic PDH enzyme activity was shown to decrease in intestinal tissues in groups receiving J (J + RT and J + RT + J) (Figure 6). This result indicates that J can be a chemotherapeutic agent with PDH inhibition and antitumour activity.

Acute phase response is a response to inflammation, tissue injury, neoplastic growth or immunological disorders in an organism and is characterised by metabolic and systemic changes. Inflammation, tissue damage, and infection are caused by acute phase response, and as a result of this response, acute phase proteins (APPs) are synthesised in the liver. The functions of APPs include Hb binding to prevent iron loss, free radical scavenging to prevent oxidation of lipids, binding bacterial components, carrying cholesterol, and preventing microbial growth. The concentration of APPs increases rapidly in the case of infection, tissue damage, and neoplastic growth. Total protein, albumin, and globulin are some of the APPs^38^^,^^39^. Albumin is one of the most frequently used biochemical parameters to assess nutritional status^40^. In cases of malnutrition, albumin levels decrease. In our study, no statistically significant difference was observed among groups in total protein, albumin and globulin levels. The main critical objectives for radiation are the complex structure of lipids in the structure of cell membrane, metabolically important enzymes and nucleic acids. Damage to these elements and deterioration of their function cause loss of normal function and cell death^41^. In our study, there was a statistically significant increase in ALT (*p*< .001) and AST (*p*< .05) liver enzymes in the RT group comparing no RT and RT groups. The possible cause of this increase is abdominopelvic irradiation as seen in the histopathological findings in the liver connected to damage. Although there was a statistically significant increase in ALT level between no RT and J + RT groups (*p*< .001), there was no statistically significant difference in the no RT and J + RT + J groups (*p*> .05). Although there was a statistically significant increase in AST level in the J groups among no RT and J + RT and J + RT + J groups (*p*< .05). There was no statistically significant difference between the groups with respect to GGT level. Biochemical results support the protective effect of J based on ALT and AST enzyme findings (Table 6).

## Conclusions

In summary, J has been shown to have a protective effect on radiation-induced GI damage in our study. First, the use of J for experimentally induced GI tissue damage showed a protective effect on weight loss. At the same time, we showed histopathologically that it prevents the exacerbation of tissue damage. Immunohistochemical results indicated that J was a PDH inhibitor. Some biochemical results have been supporting the protective effect of J. When all findings were evaluated together, improvement in the J + RT + J group was better than the J + RT group. Therefore, it was concluded that it may be more useful to administer J before and after RT for radioprotection. Lastly, all these results suggest that J may have a promising role in future treatment of GI radiation damage.
